# ChREBP**β** is dispensable for the control of glucose homeostasis and energy balance

**DOI:** 10.1172/jci.insight.153431

**Published:** 2022-02-22

**Authors:** Emeline Recazens, Geneviève Tavernier, Jérémy Dufau, Camille Bergoglio, Fadila Benhamed, Stéphanie Cassant-Sourdy, Marie-Adeline Marques, Sylvie Caspar-Bauguil, Alice Brion, Laurent Monbrun, Renaud Dentin, Clara Ferrier, Mélanie Leroux, Pierre-Damien Denechaud, Cedric Moro, Jean-Paul Concordet, Catherine Postic, Etienne Mouisel, Dominique Langin

**Affiliations:** 1 Institute of Metabolic and Cardiovascular Diseases (I2MC), Equipe MetaDiab, University of Toulouse, INSERM, University of Toulouse III - Paul Sabatier (UPS), Toulouse, France.; 2 University of Paris, Cochin Institute, CNRS, INSERM, Paris, France.; 3 Toulouse University Hospital Center, Biochemisty Laboratory, Toulouse, France.; 4 Genome Structure and Instability Laboratory, INSERM, CNRS, National Museum of Natural History, Paris, France.; 5 Academic Institute of France (IUF), Paris, France.

**Keywords:** Metabolism, Adipose tissue, Carbohydrate metabolism, Obesity

## Abstract

Impaired glucose metabolism is observed in obesity and type 2 diabetes. Glucose controls gene expression through the transcription factor ChREBP in liver and adipose tissues. *Mlxipl* encodes 2 isoforms: ChREBPα, the full-length form (translocation into the nucleus is under the control of glucose), and ChREBPβ, a constitutively nuclear shorter form. ChREBPβ gene expression in white adipose tissue is strongly associated with insulin sensitivity. Here, we investigated the consequences of ChREBPβ deficiency on insulin action and energy balance. ChREBPβ-deficient male and female C57BL6/J and FVB/N mice were produced using CRISPR/Cas9-mediated gene editing. Unlike global ChREBP deficiency, lack of ChREBPβ showed modest effects on gene expression in adipose tissues and the liver, with variations chiefly observed in brown adipose tissue. In mice fed chow and 2 types of high-fat diets, lack of ChREBPβ had moderate effects on body composition and insulin sensitivity. At thermoneutrality, ChREBPβ deficiency did not prevent the whitening of brown adipose tissue previously reported in total ChREBP-KO mice. These findings revealed that ChREBPβ is dispensable for metabolic adaptations to nutritional and thermic challenges.

## Graphical abstract

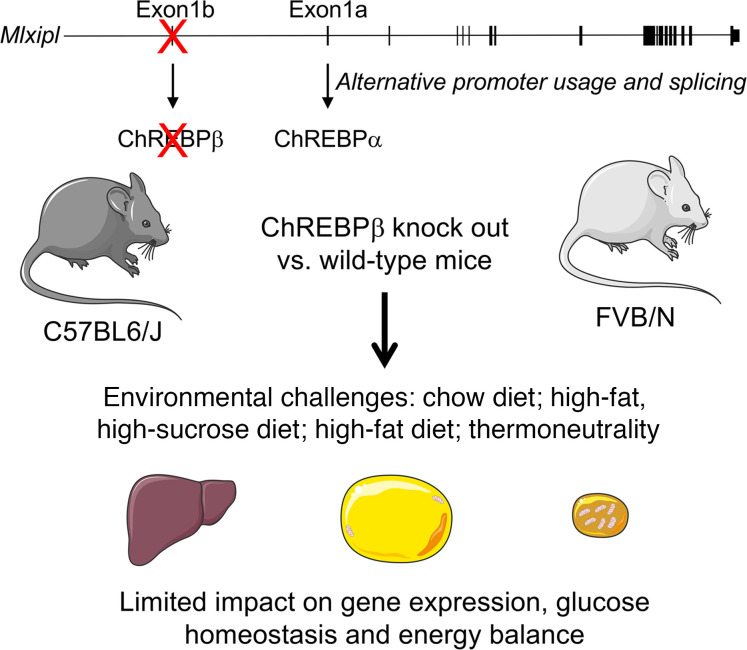

## Introduction

In mammals, ChREBP (carbohydrate responsive element binding protein) is a transcription factor mediating the response to dietary carbohydrates (1, 2). ChREBP is highly expressed in the liver and adipose tissues, where it acts as a master regulator of lipid synthesis. The ChREBP protein contains several functional domains, including nuclear import and export signals and a polyproline region in the C-terminal region. The C-terminal region also contains a DNA-binding basic helix-loop-helix/Zip domain, responsible for ChREBP transcriptional activity through heterodimerization with MLX (Max-like protein X) (3). Serial deletions of the ChREBP protein led to the identification of an evolutionarily conserved domain, the glucose-sensing module (GSM), composed of a low-glucose inhibitory domain (LID) and a transactivation domain called glucose-response activation conserved element (GRACE) (4). The glucose responsiveness of ChREBP is mediated by a dynamic intramolecular inhibition between LID and GRACE, where low glucose concentrations restrain the transcriptional activity of GRACE through the LID, while high glucose releases this inhibition.

In 2012, a variant of ChREBP, termed ChREBPβ, transcribed from an alternative promoter located upstream of the previously known promoter adjacent to exon 1a, was identified in mouse white adipose tissue (5). The ChREBPβ transcript undergoes splicing from exon 1b to exon 2; translation begins at exon 4, leading to a shorter protein (687aa for ChREBPβ vs. 864aa for the full-length ChREBP renamed ChREBPα). Interestingly, a feed-forward mechanism has been suggested that places ChREBPβ under the transcriptional control of ChREBPα. In this scenario, ChREBPα is first activated by glucose metabolism and, in turn, stimulates ChREBPβ expression through a carbohydrate responsive element (ChoRE) in exon 1b, thus amplifying the response to glucose (5). From a structural point of view, ChREBPβ lacks most of the LID domain. Its transcriptional activity has been reported to be independent of glucose concentrations and 20-fold higher than that of ChREBPα (5). In addition, ChREBPβ lacks the N-terminal domain, which, by interacting with 14-3-3 proteins, involves cytosolic sequestration and therefore protein stability (6).

A central role for adipose ChREBPβ isoform in insulin sensitivity was suggested in different models of obesity and glucose intolerance where expression of ChREBPβ in white adipose tissue strongly correlates with lipogenic activity and systemic insulin sensitivity (5, 7–9). High-fat feeding results in early downregulation of white adipose tissue ChREBPβ during development of obesity (5, 9). In mouse and human adipose tissues, ChREBPβ gene expression is more closely associated with insulin sensitivity than ChREBPα gene expression (5, 7, 9–12). However, variations in ChREBPβ protein levels have not been reported so far.

The differences in induction pattern and transcriptional activity and the link with insulin sensitivity suggest distinct physiological roles of the 2 ChREBP isoforms. While studies suggest that ChREBPβ expression reflects total ChREBP lipogenic activity in white adipose tissue and the liver, the specific contribution of ChREBPβ to metabolic adaptations in vivo is not known (5, 13). Here, we investigated the consequences of ChREBPβ deficiency on insulin action and energy balance. The lack of ChREBPβ was studied in male and female mice from 2 genetic backgrounds, C57BL6/J and FVB/N. Whole-body and tissue characterizations were performed in mice fed chow and high-fat diets. The effect of housing temperature was also studied.

## Results

**Figure 1:**
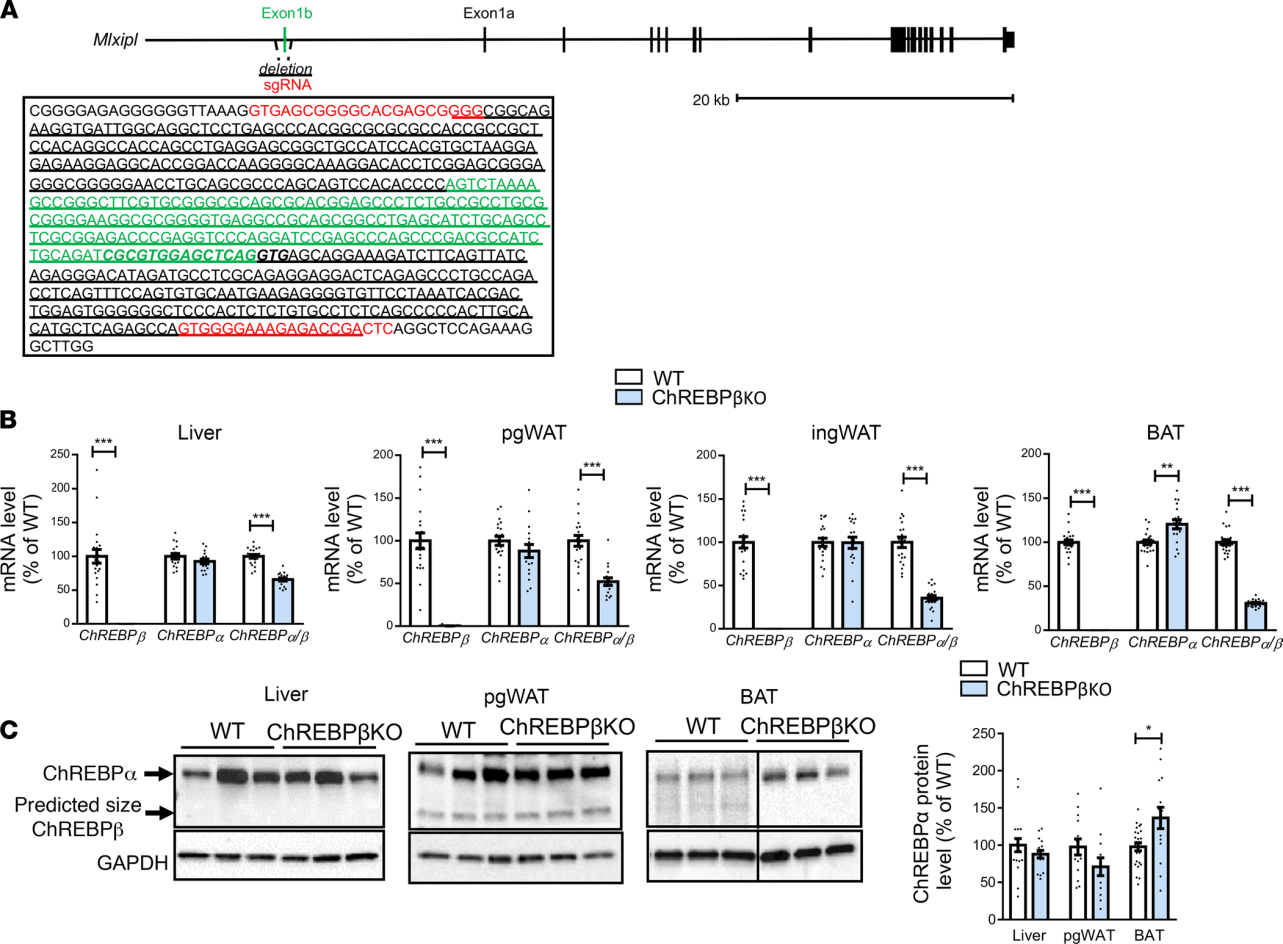
Generation of ChREBPβ-deficient mice. (**A**) Structure of the *Mlxipl* gene. sgRNA sequences are shown in red. Exon 1b sequence is shown in green. Sequence of the carbohydrate responsive element (ChoRE) is italicized and bolded. Deleted sequence is underlined. (**B**) mRNA levels of ChREBP isoforms in liver, perigonadal white adipose tissue (pgWAT), inguinal WAT (ingWAT), and interscapular brown adipose tissue (BAT) of refed C57BL6/J male (*n* = 16–21) mice. (**C**) Representative Western blots and quantitation of ChREBP protein levels in liver, pgWAT, and BAT of refed C57BL6/J male mice (*n* = 12–24). Data are mean ± SEM. Statistical analysis was performed using Mann-Whitney test. *P < 0.05, **P < 0.01, ***P < 0.001.

We used CRISPR/Cas9-mediated gene editing, which recently emerged as an efficient technology to generate gene KO mouse models to produce ChREBPβ-deficient mice (14). Compared with homologous recombination in embryonic stem cells, targeted gene editing in one-cell embryos may be more rapid. Electroporation of fertilized oocytes was performed using 2 guide RNAs targeting ChREBPβ-specific exon (Figure 1A) complexed to purified Cas9 protein. The protocol proved highly efficient: one-third to one-half of F0 mice had expected deletion (Supplemental Table 1A; supplemental material available online with this article; https://doi.org/10.1172/jci.insight.153431DS1). Counting from the electroporation day, the first experimental groups composed of F2 mice were obtained in 7 months. ChREBPβ deficiency was induced in C57BL6/J and FVB/N strains to assess the influence of the genetic background on the phenotype. We derived for each of the strains 2 transgenic lines and observed no phenotypic differences between the lines (data not shown). Mice were born at the expected Mendelian ratio. The number of pups per litter was similar in WT and ChREBPβ-deficient mice (Supplemental Table 1B). Examination of pup appearance and growth during lactation and the weaning period did not reveal any gross defect due to the absence of ChREBPβ.

Gene expression analyses in tissues expressing ChREBP showed complete lack of ChREBPβ mRNA in male and female mice (Figure 1B and Supplemental Figure 1, A–C). Quantitation of ChREBP isoform mRNA levels showed that ChREBPβ contributed more to total ChREBP transcripts in adipose tissues than in the liver (Figure 1B and Supplemental Figure 1, A–D). In C57BL6/J and FVB/N male but not female mice with ChREBPβ deficiency, mRNA and protein levels of ChREBPα were increased in brown adipose tissue and unchanged in other tissues (Figure 1, B and C, and Supplemental Figure 1, A–C and E). In male and female mice of the 2 strains, induction of ChREBPβ gene expression during refeeding with glucose in food and water was more pronounced in brown adipose tissue than in white fat pads (Supplemental Figure 1D). The lowest induction was observed in the liver. These profiles were observed in male and female mice from the 2 genetic strains. The rank order of induction is consistent with earlier data suggesting higher induction of ChREBPβ in white fat than in the liver and with the importance of glucose uptake in brown adipose tissue (5, 15–17). In contrast, ChREBPα mRNA levels showed the highest induction in perigonadal fat and the liver. Of note, we failed in detecting a specific band corresponding to ChREBPβ protein (Figure 1C).

**Figure 2:**
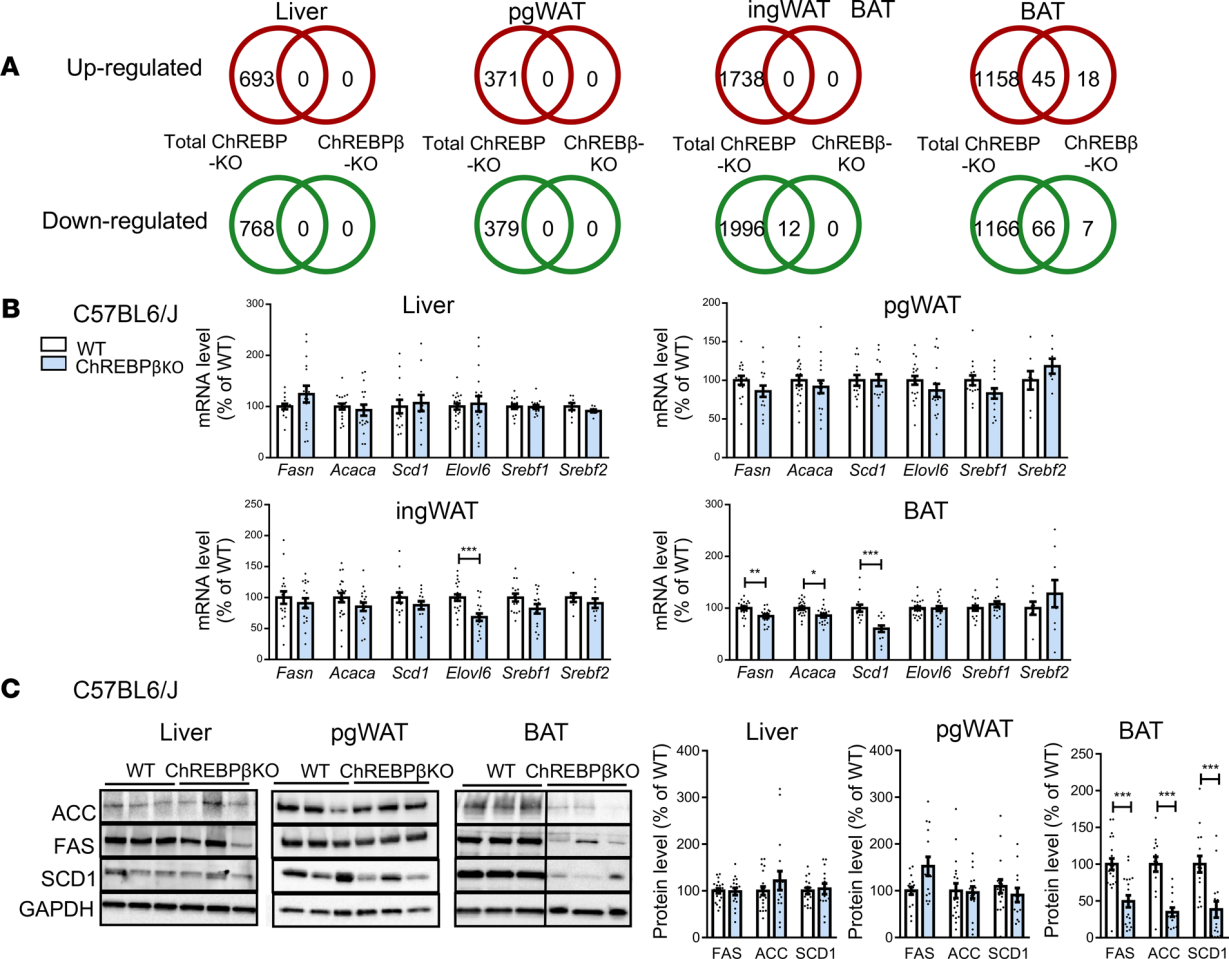
Gene expression profiles in male ChREBPβ-deficient mice. (**A**) Number of regulated genes in the absence of ChREBPβ (ChREBPβ KO) or of ChREBPα and β isoforms (total ChREBP KO) compared with WT in liver, perigonadal white adipose tissue (pgWAT), inguinal WAT (ingWAT), and interscapular brown adipose tissue (BAT) of C57BL6/J refed male mice (*n* = 6–8 per group). (**B**) mRNA levels of de novo lipogenesis genes in liver, pgWAT, ingWAT, and BAT of C57BL6/J mice (*n* = 7–21). (**C**) Representative Western blots and quantitation of de novo lipogenesis proteins in liver, pgWAT, ingWAT, and BAT of C57BL6/J refed male mice (*n* = 12–21). Representative Western blots for pgWAT here and in Figure 1C are from the same experiment. Data are mean ± SEM. Statistical analysis was performed using Mann-Whitney test. *P < 0.05, **P < 0.01, ***P < 0.001.

To assess the transcriptional consequences of the lack of ChREBPβ in target tissues, transcriptomic analysis using DNA microarrays was performed in parallel in white (perigonadal and inguinal depots) and brown (interscapular depot) adipose tissues and in the liver of C57BL6/J male mice with deficiency in ChREBPβ or in ChREBPα and β isoforms (*Mlxipl*-null mice, here referred to as total ChREBP-KO mice) (18) (Figure 2A). Unexpectedly, in the liver and white adipose tissues, no significant change in gene expression was observed when ChREBPβ was lacking, whereas, as expected, multiple genes and pathways were regulated in total ChREBP-KO mice (Figure 2A and Supplemental Figure 2A). In brown adipose tissue, 63 and 73 genes were, respectively, upregulated and downregulated in ChREBPβ-deficient versus WT mice (Figure 2A). A majority of these genes were also regulated in brown adipose tissue of total ChREBP-KO mice. Eighteen upregulated and 7 downregulated genes may be specific to the absence of ChREBPβ compared with lack of the 2 ChREBP isoforms (Figure 2A and Supplemental Table 2). Of note, the number of genes regulated in brown adipose tissue of ChREBPβ-deficient mice represents less than 5% of the number of genes regulated in brown adipose tissue of total ChREBP-KO mice (Figure 2A). Because ChREBP is known to be a master regulator of de novo lipogenesis, we further probed the effect of ChREBPβ deficiency through measurements of cognate mRNA and protein levels in the liver and adipose tissues of male and female C57BL6/J and FVB/N mice. In mice refed after a fasting period to favor induction of de novo lipogenesis, ChREBPβ deficiency induced impairment in mRNA and protein expression chiefly in brown adipose tissue (Figure 2, B and C, and Supplemental Figure 2, B–D). In fasting conditions, lack of ChREBPβ affected expression of de novo lipogenesis genes mainly in brown adipose tissue, suggesting that ChREBPβ may play a role in the maintenance rather than induction of gene expression by glucose (Supplemental Figure 3, A and B). The number of genes and magnitude of mRNA level changes in brown adipose tissue are much lower in mice lacking ChREBPβ than in total ChREBP-KO mice. Altogether, the data suggest that ChREBPβ plays no significant role in white adipose tissue and liver gene expression.

**Figure 3:**
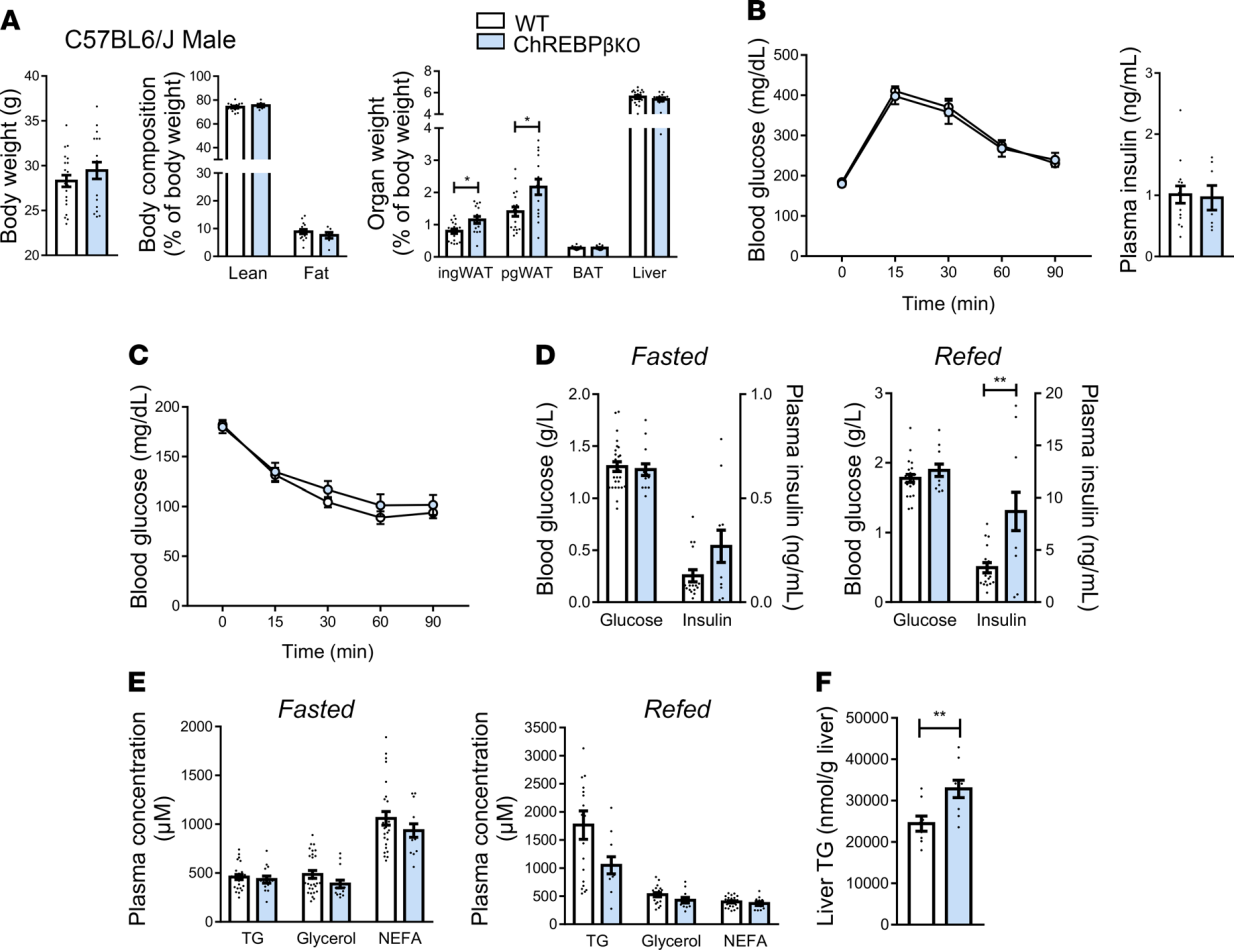
Body composition and glucose homeostasis in male ChREBPβ-deficient mice. (**A**) Body weight, lean and fat mass, and organ weight in male C57BL6/J (*n* = 7–18) mice. (**B**) Glucose tolerance test and blood insulin levels 15 minutes after injection during glucose tolerance tests (*n* = 7–18). (**C**) Insulin tolerance test (*n* = 9–18). (**D**) Blood glucose and insulin levels after 24 hours of fasting (left panels) and 18 hours of refeeding (right panels) (*n* = 11–17). (**E**) Blood lipid levels after 24 hours of fasting (left panels) and 18 hours of refeeding (right panels) (*n* = 11–17). (**F**) Liver triglyceride (TG) levels after 18 hours of refeeding (*n* = 8–9). NEFA, nonesterified fatty acid. Data are mean ± SEM. Statistical analysis was performed using Mann-Whitney test (**A**, **B**, and **D–F**) or 2-way ANOVA with Šídák’s post hoc test (**B** and **C**). *P < 0.05, **P < 0.01.

Given that combined deficiency in ChREBPα and β results in alterations of metabolic traits, we next addressed the functional consequences of the lack of ChREBPβ on energy balance and glucose homeostasis. In mice fed a chow diet, body weight and lean and fat mass were not different between genotypes in male and female C57BL6/J and FVB/N mice (Figure 3A, Supplemental Figure 4A, and Supplemental Figure 5, A and B). The higher weight of perigonadal and inguinal white adipose tissues was only observed in C57BL6/J male mice (Figure 3A). No difference was observed during insulin and glucose tolerance tests except for a genotype effect in female C57BL6/J mice with a maximum of 20% difference in glycemia during the insulin tolerance test (Figure 3, B and C, Supplemental Figure 4, B and C, and Supplemental Figure 5, C and D). Regarding the latter group, the difference between the 2 curves was no longer significant when data were expressed as a percentage of glucose levels at the beginning of the test (data not shown). Concerning plasma parameters, glucose and insulin levels in fasting and refeeding conditions were similar except for the higher plasma insulin levels seen in refeeding conditions in male and female C57BL6/J mice (Figure 3D and Supplemental Figure 4D, and Supplemental Figure 5, E and F). There were no marked genotype differences in blood lipid parameters in male and female mice of the 2 genetic strains (Figure 3E, Supplemental Figure 4E, and Supplemental Figure 5, G and H). No difference was seen in liver triglyceride levels except for male C57BL6/J mice (Figure 3F, Supplemental Figure 4F, and Supplemental Figure 5, I and J). These data showed that ChREBPβ is not required to maintain body composition and glucose homeostasis in mice fed a chow diet.

**Figure 4:**
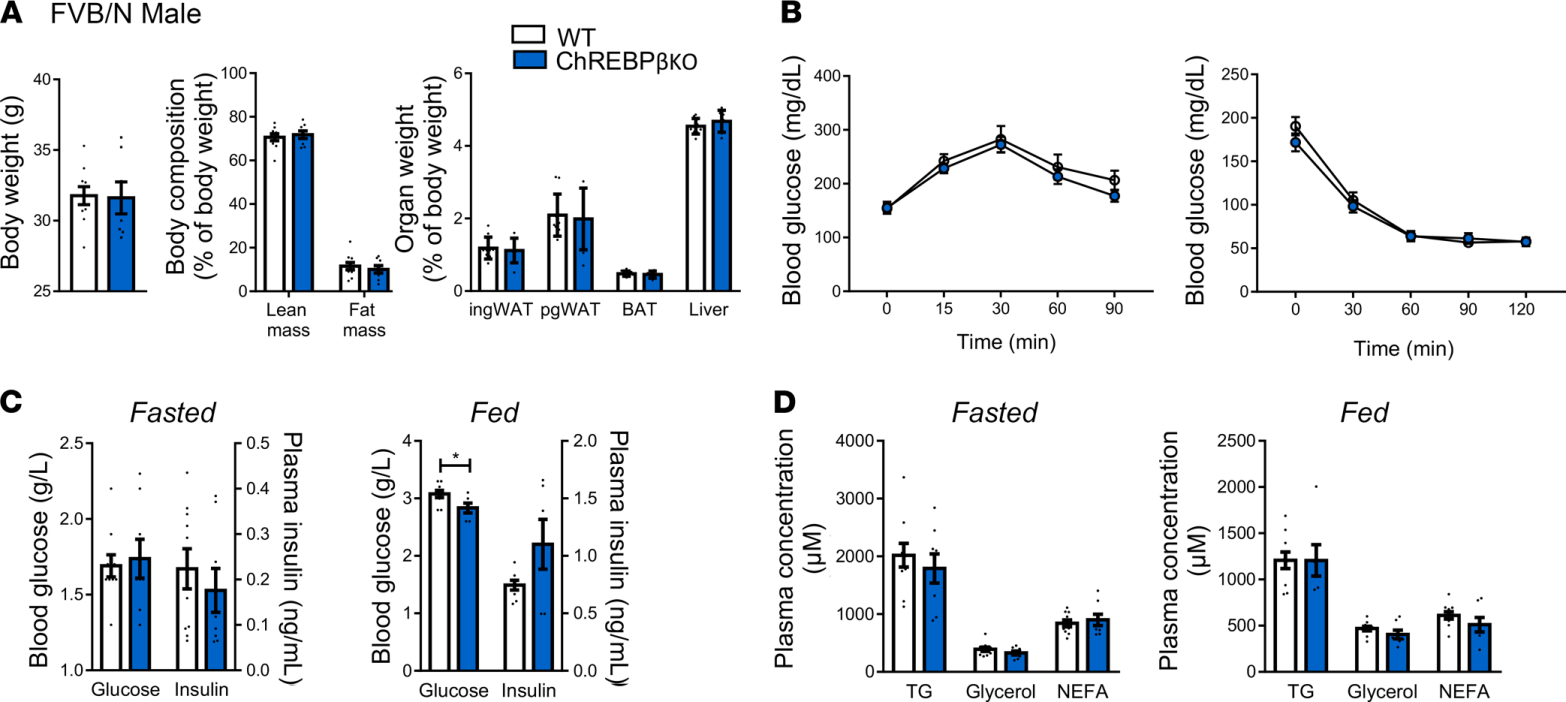
Response of ChREBPβ-deficient mice to high-fat, high-sucrose diet. (**A**) Body weight, lean and fat mass, and organ weight in male FVB/N (*n* = 7–14) mice fed high-fat, high-sucrose diet. (**B**) Glucose (left panels) and insulin (right panels) tolerance tests (*n* = 8–14). (**C**) Blood glucose and insulin levels after 24 hours of fasting (left panels) and 18 hours of refeeding (right panels) (*n* = 6–14). (**D**) Blood lipid levels after 24 hours of fasting (left panels) and 18 hours of refeeding (right panels) (*n* = 6–10). Data are mean ± SEM. Statistical analysis was performed using Mann-Whitney test (**A**, **C**, and **D**) or 2-way ANOVA with Šídák’s post hoc test (**B**). *P < 0.05.

**Figure 5:**
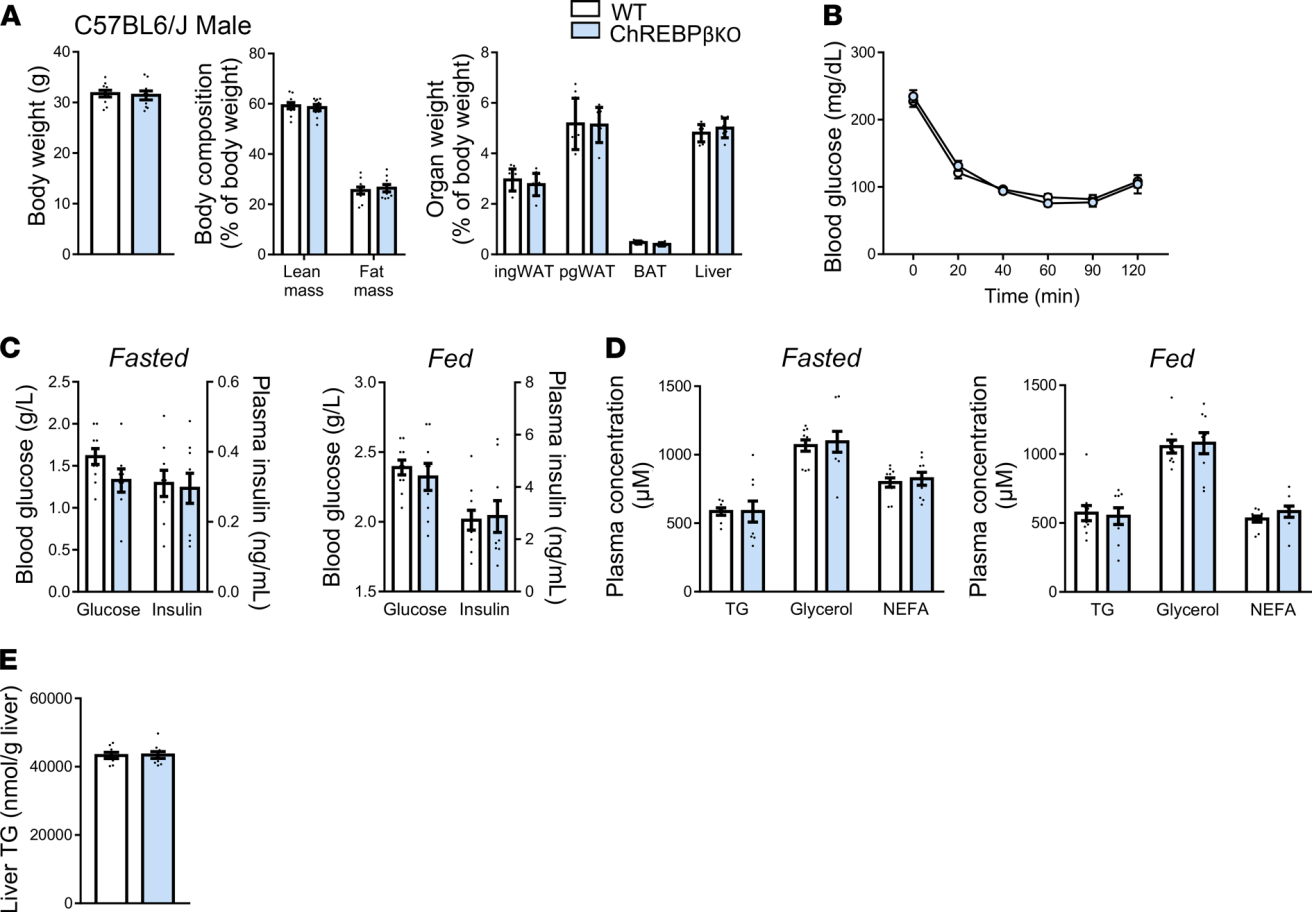
Response of ChREBPβ-deficient mice to high-fat (45%) diet. (**A**) Body weight, lean and fat mass, and organ weight in male C57BL6/J mice (*n* = 9–10). (**B**) Insulin tolerance test (*n* = 9–10). (**C** and **D**) Blood glucose, insulin (**C**), and lipid (**D**) levels in fasted (left panels) and fed (right panels) conditions (*n* = 8–10). (**E**) Liver triglyceride (TG) levels after 18 hours of refeeding (*n* = 9). NEFA, nonesterified fatty acid. Data are mean ± SEM. Statistical analysis was performed using Mann-Whitney test (**A** and **C–E**) or 2-way ANOVA with Šídák’s post hoc test (**B**).

To unravel the potential role of ChREBPβ in pathophysiological conditions, ChREBPβ-deficient mice were subjected to nutritional and thermic challenges. Feeding a high-fat, high-sucrose diet (32% fat, 51% carbohydrates containing 50% sucrose) for 12 weeks resulted in lower body weight and fat mass in female C57BL6/J mice but not in male and female FVB/N mice lacking ChREBPβ compared with WT littermates (Figure 4A and Supplemental Figure 6, A and B). Glucose and insulin tolerance tests showed no genotype effect (Figure 4B and Supplemental Figure 6, C and D). Blood glucose, insulin, lipid, and liver triglyceride levels in the 2 genotypes were similar in fasting and refeeding conditions for most of the measurements (Figure 4, C and D, and Supplemental Figure 6, E–J). The few significant differences were not consistently found in the various nutritional conditions or sex and genetic backgrounds investigated in this work. Feeding male C57BL6/J mice a diet with 45% fat for 12 weeks resulted in no difference in body composition and insulin tolerance according to genotype (Figure 5, A and B). Plasma glucose and insulin as well as blood lipid levels were similar in fasting and refeeding conditions (Figure 5, C and D). No difference was seen in liver triglyceride levels (Figure 5E). These phenotypic data on body composition and insulin sensitivity are in agreement with the limited impact of ChREBPβ deficiency on gene expression profiles.

**Figure 6:**
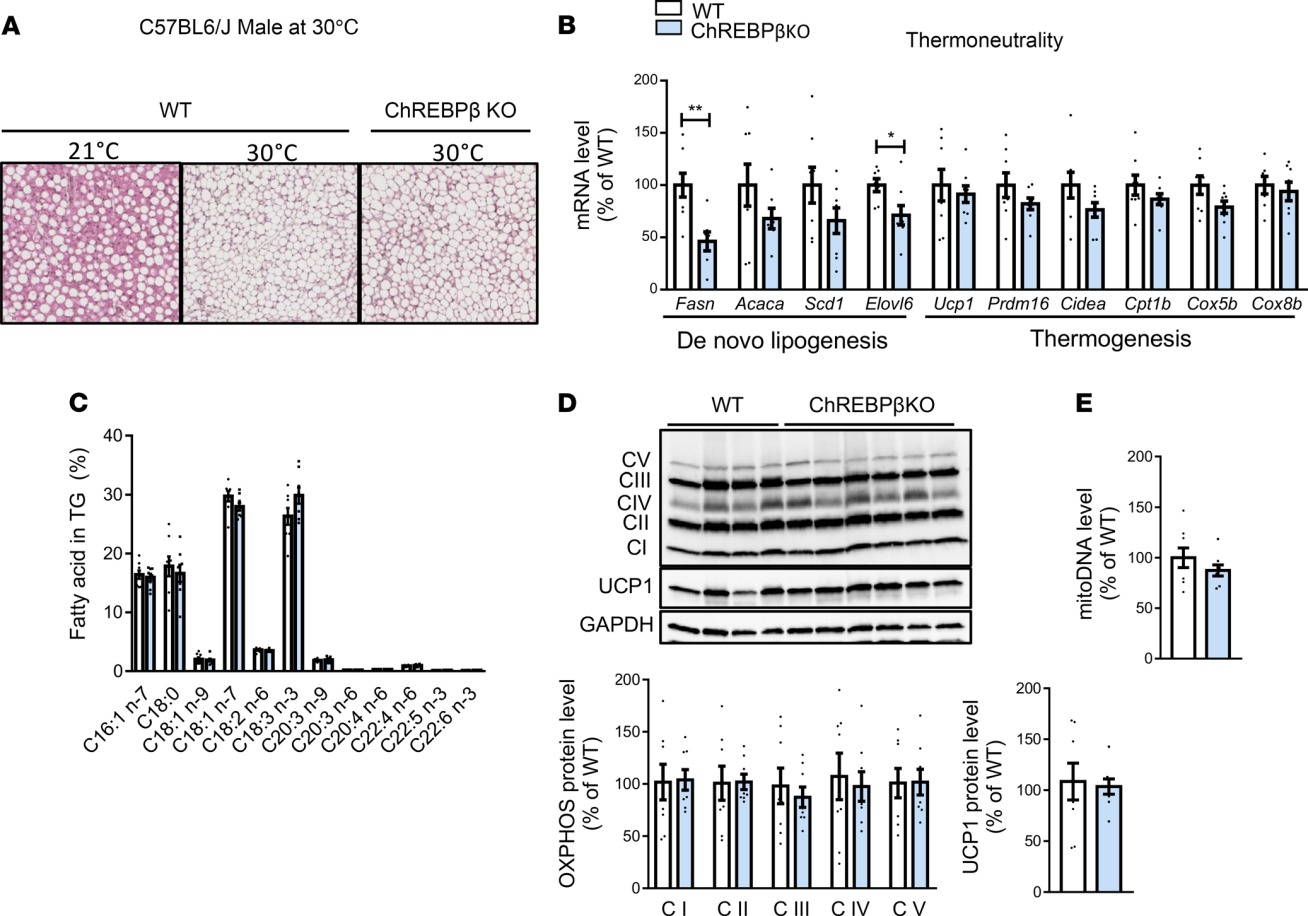
Response of ChREBPβ-deficient mice to thermic challenges. (**A–E**) Representative H&E staining (**A**), mRNA levels of de novo lipogenesis and thermogenic genes (**B**), fatty acid profile in triglycerides (TG) (**C**), OXPHOS and UCP1 protein levels (**D**), and mitochondrial DNA (mitoDNA) content (**E**) in interscapular brown adipose tissue of C57BL6/J male mice adapted to thermoneutrality for 6 weeks (*n* = 7–9). Data are mean ± SEM. Statistical analysis was performed using Mann-Whitney test. *P < 0.05, **P < 0.01.

The impairment of de novo lipogenesis in brown adipose tissue of mice with total ChREBP deficiency has been reported to prevent the decrease in mitochondrial oxidative capacity and tissue whitening induced by thermoneutral adaptation (19). Because the highest level of ChREBPβ mRNA expression was observed in brown adipose tissue (Supplemental Figure 1D), we investigated whether ChREBPβ deficiency led to a similar adaptation. WT and ChREBPβ-deficient male C57BL6/J mice were transferred from a housing temperature of 21°C to a thermoneutral condition of 30°C over 6 weeks. Histological analyses revealed similar transition to white adipocyte–like appearance of interscapular brown adipose tissue in WT and ChREBPβ-deficient littermates (Figure 6A). As observed at a housing temperature of 21°C (Supplemental Figure 2D), gene expression of *Fasn* and *Elovl6* was lower in ChREBPβ-deficient mice at 30°C (Figure 6B). However, the changes in de novo lipogenesis gene expression did not translate into differences in the profile of fatty acids composing brown adipose tissue triglycerides (Figure 6C). Genes involved in thermogenesis showed similar expression in the 2 genotypes (Figure 6B). Accordingly, OXPHOS and UCP1 protein levels and mitochondrial DNA content were comparable in the brown fat of WT and ChREBPβ-deficient mice (Figure 6, D and E). Altogether, our data revealed that ChREBPβ deficiency did not influence the involution of brown adipose tissue observed during adaptation to a thermoneutral environment.

## Discussion

The *Mlxipl* gene encodes 2 isoforms. ChREBPα contains the functional domains governing DNA binding, modulation of transactivation, and the highly regulated intracellular trafficking between the cytosol and the nucleus (1, 2). Generated from alternative promoter usage and splicing, ChREBPβ is a shorter protein lacking the N-terminal regions involved in intracellular trafficking and the control of transactivation (5). In human and mouse adipocytes, induction of ChREBPβ gene expression in conditions of increased glucose transport is much higher than for ChREBPα, suggesting an important role for this isoform (5, 8, 12). Opposite to our expectations, we observed mild consequences of a lack of ChREBPβ on ChREBP target gene expression, body composition, and whole-body glucose homeostasis.

CRISPR/Cas9-mediated gene editing recently emerged as an efficient technology to generate gene KO mouse models (14). As homologous recombination in embryonic stem cells is replaced by targeted gene editing in one-cell embryos, it proves remarkably rapid in generating multiple lines of KO mice. Electroporation of fertilized oocytes as shown here further simplifies and speeds up the process with remarkable efficiency. It allows generation of transgenic lines on various genetic backgrounds without costly mouse- and time-consuming backcrossing and favors better compliance to the 3R principles (Replacement, Reduction, and Refinement). Most gene KO models in the metabolism field have been generated in the C57BL6 strain. Various inbred strains show differences in whole-body and tissue responses, so the consequences of gene deficiency may vary between strains and alter conclusions on phenotypes associated with genetic ablation, as exemplified by *Lep*^ob/ob^ mouse studies (20). Therefore, we produced ChREBPβ-deficient mice on C57BL6/J and FVB/N backgrounds because the 2 strains show differences in terms of body composition and metabolic responses to environmental challenges that are dependent on sex (21–23). The consequences of a lack of ChREBPβ were similar in the 2 strains in both sexes, pointing at a moderate impact on systemic insulin action and energy balance.

In male and female mice of the 2 strains, ChREBPβ expression was strongly induced when a fasting period was followed by refeeding. The exquisite sensitivity of ChREBPβ gene expression to glucose, constitutive nuclear localization, and association with insulin sensitivity in humans has been considered as supporting a role for ChREBPβ being a much more potent transcriptional activator than ChREBPα (5, 10–12). However, the data of the current study in ChREBPβ-deficient mice do not support such a role. Deficiency in both ChREBPα and β had a profound impact on gene expression in the tissues tested, whereas parallel DNA microarray analyses showed an impact of ChREBPβ deficiency restricted to a few genes in brown fat. Direct measurements of known targets of ChREBP involved in de novo lipogenesis confirmed a limited impact at mRNA and protein levels, chiefly observed in brown adipose tissue. Our in vivo data are coherent with a model where ChREBPβ is a weak transactivator as suggested in vitro (24, 25). In the context of massive overexpression, ChREBPβ, which can freely enter the nucleus may, however, exert a transactivation capacity. However, as reported by others, we failed to detect the ChREBPβ protein through Western blot analysis in mouse tissues, suggesting very low levels of endogenous expression in vivo (9, 26).

The limited impact of ChREBPβ deficiency was also seen on energy balance and insulin sensitivity. Combined deficiency in ChREBPα and β results in metabolic alterations that are not observed when only ChREBPβ is lacking. Total ChREBP-KO mice have lower amount of gonadal white adipose tissue than WT littermates (27). Phenotypic differences between global ChREBP and ChREBPβ-selective deficiency are exacerbated when investigating glucose homeostasis and insulin sensitivity. The impaired glucose and insulin tolerance reported in total ChREBP-KO mice was not found in ChREBPβ-deficient mice (28). Whereas adipose- and liver-specific ablations of both ChREBPα and β alter insulin sensitivity in mice fed chow and high-fat diets, we found no modification of insulin and glucose tolerance in ChREBPβ-deficient mice regardless of sex, genetic strain, or diet (29, 30).

Because the highest level of ChREBPβ mRNA expression was observed in brown adipose tissue, we performed further analyses on this tissue. ChREBPβ deficiency did not influence the involution of brown adipose tissue observed during adaptation to a thermoneutral environment. In contrast, the absence of ChREBPα and β results in inhibition of fatty acid synthesis in brown adipose tissue, preventing whitening of the tissue (19). The impairment of de novo lipogenesis in mice with global ChREBP deficiency results in preservation of mitochondrial mass and function during transition from a housing temperature of 21°C to 30°C. Brown fat from ChREBPβ-deficient mice showed similar fatty acid profiles, triglyceride amount, and mitochondrial protein and DNA levels as WT littermates, indicating unaltered involution of brown adipose tissue at thermoneutrality.

This study has limitations. For the in vivo investigation of ChREBPβ deficiency, ablation was performed in all tissues expressing the transcription factor. It cannot, therefore, be excluded that different actions in tissues expressing ChREBPβ result in compensatory mechanisms and the mild phenotype reported here. However, experiments performed in multiple genetic, sex, and environmental conditions potentially engaging ChREBPβ did not support a major role in these adaptations. After other nutritional challenges, such as high fructose or alcohol intake, it may be envisaged that ChREBPβ contributes more to ChREBP transcriptional activity than in the conditions reported here (13, 31, 32). Finally, identification of factors regulating ChREBPβ protein stability may be required to pave the way for future in vivo investigation of ChREBPβ.

In conclusion, we showed that ChREBPβ, a shorter product of the *Mlxipl* gene, is dispensable in vivo for metabolic adaptations to nutritional and thermic challenges. ChREBPα exerts the predominant role in these adaptations.

## Methods

### Mouse strains and environmental challenges.

C57BL6/J and FVB/N male and female ChREBPβ-deficient and WT littermate mice (Janvier Labs) were used. Mice from several litters were used in each protocol to avoid litter-to-litter variation. Mice were housed at 21°C with food and water provided ad libitum and maintained on a 12-hour light/12-hour dark cycle(8 am–8 pm). Mice were fed either chow (Ssniff V1534); a high-fat, high-sucrose diet (Ssniff E15778-3447); or a 45% high-fat diet (Ssniff E15744-34). In fasting refeeding experiments, mice were fasted for 24 hours followed by refeeding with a diet used prior to fasting and drinking water containing 20% glucose for 18 hours. During thermoneutrality experiments, mice were placed in vented animal cabinets (Noroit A-box EP-20) for 6 weeks at 30°C.

### Generation of ChREBPβ-deficient mice.

One-cell embryos from 2 genetic backgrounds, C57BL6/J and FVB/N (Janvier Labs), were used. Five- to seven-week-old female mice were super-ovulated by i.p. administration of 5 IU of pregnant mare serum gonadotropin (Centravet), followed by an additional i.p. injection of 5 IU human chorionic gonadotropin 42 to 48 hours later (Centravet). Super-ovulated females were mated with adult males and euthanatized at 0.5 dpc. Oviduct were dissected and the ampulla nicked to release zygotes associated with surrounding cumulus cells into a dish of hyaluronidase (Merck-Sigma) in M2 solution (300 μg/mL, Merck-Sigma) under a stereomicroscope. Zygotes were washed 3 times in M2 medium to remove cumulus cells. Zygotes were kept in KSOM (potassium simplex optimization medium) (Merck-Sigma) in a water-jacketed CO_2_ incubator (5% CO_2_, 37°C) until electroporation.

In silico analysis was performed using CRISPOR freeware (http://crispor.tefor.net) (33). A 991 bp sequence containing the 171 bp of *Mlxipl* exon 1b was chosen as target (Figure 1A). Guide RNA (sgRNA) was designed both upstream and downstream of exon 1b. sgRNAs were produced by T7 Hiscribe transcription kit (NEB) and purified by EZNA microelute RNA cleanup kit (Omega Bio-Tek), eluted in approximately 25 μL of RNase-free water, and stored at –80°C. Efficacies of sgRNAs were tested in mouse embryonic fibroblasts using 0.5 μg of SpyCas9-NLS mRNA and 0.5 μg of sgRNA to be tested. After genomic DNA extraction, T7 endonuclease tests were performed on upstream and downstream amplicons.

To remove the entire sequence of *Mlxipl* exon 1b, SpyCas9-NLS was complexed with 2 sgRNAs (Figure 1A). Aliquots of sgRNA were denaturated at 80°C for 2 minutes and then put on ice for 2 minutes before adding the protein. The complex was performed at 1–1.5:1 (sgRNA/Cas9 protein) molar stoichiometry. Cas9 protein and sgRNA were incubated for 10 minutes at room temperature and kept on ice during electroporation.

Repeated pulses of electroporation were delivered by a NEPA 21 electroporator (Sonidel Ltd.) to first create pores in the *zona pellucida* and then favor the intracellular entry of ribonucleoproteins (34). A glass chamber with platinum plate electrodes (CUY520P5, Sonidel Ltd.) was filled with 50 μL of Opti-MEM I reduced serum medium (Thermo Fisher Scientific) or with ribonucleoprotein-containing medium. Between 23 and 48 fresh embryos were aligned in the chamber, taking care to avoid contacts between zygotes and electrodes. Impedance was measured and maintained between 0.5 and 0.54 kΩ by liquid volume adjustment (reducing volume increases impedance). Four “poring pulses” were applied (250 V, 1 ms, interval 50 ms, 10% voltage decay; positive polarity), followed by 5 “transfer pulses” (20 V, 50 ms, interval 50 ms, 40% voltage decay; alternating positive and negative polarity). Zygotes were then transferred into KSOM medium and kept in an incubator until reimplantation into the oviduct of Swiss-CD1 (Janvier Labs) pseudopregnant females (10–15 embryos per female).

### Glucose and insulin tolerance tests.

For glucose and insulin tolerance tests performed around 2 pm, 1 to 2 mg/g body weight of D-(+)-glucose (Sigma-Aldrich) and 0.7 to 1.2 mU/g body weight of insulin (Lilly) were administered (i.p.) to 6-hour fasted mice according to sex, age, diet, and genetic backgrounds (35, 36). Glycemia was checked in blood from the tail vein 30 minutes before glucose injection and then at various times after glucose administration with a Glucotrend Accu-Chek Performa (Roche SAS).

### Blood analyses.

Blood was collected in EDTA tubes through retro-orbital sampling. Except for tolerance tests (see above), blood was collected around 8 am. Mouse plasma glycerol was measured by enzymatic assay (free glycerol reagent, Sigma-Aldrich); plasma nonesterified fatty acid and triglyceride levels were measured using the NEFA C kit (Wako) and TG reagent (Sigma-Aldrich), respectively. Glucose levels were measured using a glucometer (AccuCheck, Roche) or Glucose GOD FS kit (DiaSys). Plasma insulin was measured using an ultrasensitive ELISA kit (ALPCO Diagnostics).

### Gene expression analyses.

Tissues were homogenized in QIAzol buffer (Qiagen) using Precellys tissue homogenizer. Total RNA from tissues was extracted using RNeasy kit (Qiagen). RNA concentration and purity were assessed spectrophotometrically using NanoDrop (DigitalBio).

For each sample, cRNA labeled with Cyanine-3 (Cy3) was prepared from 200 ng of total RNA using the One-Color Quick Amp Labeling kit (Agilent Technologies) according to the manufacturer’s instructions, followed by RNAClean XP (Agencourt Bioscience Corporation) purification. Dye incorporation and cRNA yield were checked using Dropsense 96 UV/VIS droplet reader (Trinean). Next, 600 ng of Cy3-labeled cRNA (specific activity >6 pmol Cy3/μg cRNA) was fragmented at 60°C for 30 minutes in a reaction volume of 25 μL containing 10× Agilent fragmentation buffer and 25× Agilent blocking agent following the manufacturer’s instructions. On completion of the fragmentation reaction, 25 μL of 2× Agilent hybridization buffer was added to the fragmentation mixture and hybridized to SurePrint G3 Mouse GE v2 microarray (8X60K, design 074809, enclosed in Agilent SureHyb-enabled hybridization chambers) for 17 hours at 65°C in a rotating Agilent hybridization oven. After hybridization, microarrays were washed sequentially in wash buffer 1 (Agilent Technologies, 1 minute) and wash buffer 2 (Agilent Technologies, 37°C, 1 minute). Slides were scanned immediately after washing on an Agilent G2505C Microarray Scanner with Agilent Scan Control A.8.5.1 software. The scanned images were analyzed with Feature Extraction Software 10.10.1.1 (Agilent Technologies) using default parameters (protocol GE1_1010_Sep10 and Grid: 074809_D_F_20150624). All subsequent data analyses were done under R (www.r-project.org) using packages of Bioconductor (www.bioconductor.org). Raw data (median of pixel intensity) were imported into R using the read.maimages function from the limma package with the following weight function (assigning a weight of 1 or 0 to each spot): myfunw<-function(x) {okType<-x$ControlType==0; okFoundGreen<-x$gIsFound==1; okPos=x$gIsPosAndSignif==1; okWellAbove<- x$gIsWellAboveBG==1; as.numeric(okType & okFoundGreen & okPos & okWellAbove);}. We selected the spots with a minimal weight of 1 for 26 out of 32 microarrays or with a minimal weight of 6 per group from at least 1 experimental group for each tissue. Data were then stored in an ExpressionSet object and normalized by the quantile method using the normalize.quantiles function from the preprocessCore R library. Replicated probes on the array (identical ProbeName) were resolved by taking the median normalized signal of each set of replicated probes. Pathway analysis was performed using the ENRICHR web-based tool (37–39). Gene expression data from DNA microarray analyses reported in this study have been deposited in NCBI’s Gene Expression Omnibus (GEO GSE179564).

After treatment with DNase I (Invitrogen) and reverse transcription of 1 mg of total RNA with Multiscribe Reverse Transcriptase, real-time quantitative PCR was performed with Fast SYBR Green Master Mix or TaqMan Fast Advanced Master Mix and QuantStudio5 real-time PCR system (Thermo Fisher Scientific). HPRT (hypoxanthine-guanine phosphoribosyltransferase) mRNA was used as control to normalize gene expression. A list of primers is provided in Supplemental Table 3.

### Protein analyses.

Tissues were homogenized in RIPA buffer containing protease and phosphatase inhibitors (Sigma-Aldrich) using a Precellys homogenizer and centrifuged. Supernatants were harvested for determination of total protein concentration. Equal amounts of solubilized proteins were loaded on 4%–20% gradient SDS-PAGE gels (Bio-Rad), blotted onto nitrocellulose membranes, and incubated overnight with primary antibodies, rabbit anti-GAPDH (1:1000, CST, 2118), rabbit anti-ChREBP (1:1000, Novus, NB135), rabbit anti-SCD1 (1:1000, CST, 2794), rabbit anti-FAS (1:1000, CST, 3180), rabbit anti-ACC (1:1000, CST, 3662), mouse anti-OXPHOS (1:1000, Abcam, ab110413), and rabbit anti-UCP1 (1:1000, Abcam, ab10983). Subsequently, immunoreactive proteins were blotted with anti-rabbit or mouse HRP-labeled secondary antibodies for 1 hour at room temperature, revealed by enhanced chemiluminescence reagent (SuperSignal West Femto, Thermo Fisher Scientific), and visualized using ChemiDoc MP Imaging System; data were analyzed using ImageLab 4.2 (Bio-Rad).

### Fatty acid profiles in brown adipose tissue.

Fatty acid composition of brown adipose tissue triglycerides was determined by capillary gas chromatography. Tissue samples were homogenized in methanol/butylated hydroxy toluene (10 mg/L) and stored at –20°C until analysis. Neutral lipids (corresponding to an equivalent of 20 mg tissue) were extracted following the Folch method using chloroform/methanol (2:1 vol/vol), in the presence of the internal standard glyceryl trinonadecanoate (Sigma-Aldrich). The triglyceride fraction was isolated by thin layer chromatography on silica glass plates (Merck) using petrol ether/diethyl ether/acetic acid (80:20:1 vol/vol/vol) as the mobile phase. Separation and quantitation of fatty acid methyl esters generated by transmethylation was achieved on a GC 2030 (Shimadzu) with flame ionization detection (40).

### Hepatic triglyceride content quantitation.

First, 100 mg of crushed liver was resuspended in 500 μL of PBS and lysed using a Precellys tissue homogenizer. The suspension was diluted in absolute ethanol 1:9 and incubated 1 hour under strong shaking at room temperature. After 2 rounds of 20-second centrifugation at 492*g*, the supernatant was collected and triglyceride content was assessed as described in *Blood analyses*.

### Mitochondrial DNA content.

Brown adipose tissue was crushed into powder using a mortar and total DNA was extracted using DNeasy kit (Qiagen) according to the manufacturer’s protocol. Real-time quantitative PCR was performed as described above using *Cox1* primers for mitochondrial DNA and *Ppia* primers for nuclear DNA. Mitochondrial DNA quantitation was calculated as 2ΔCt of the mitochondrial gene *Cox1* using *Ppia* as nuclear housekeeping gene.

### Histology.

Intrascapular brown adipose tissues were fixed with 4% paraformaldehyde in PBS, dehydrated, embedded in paraffin, and cut into 7 mm sections. Sections were stained with H&E using standard protocols.

### Statistics.

Statistical analyses and graphical representations were performed using GraphPad Prism 9. Figure legends contain the description of the statistical test performed for each graphical representation as well as the number of animals used in each experiment (*n*). All results are represented as mean ± SEM. *P* values of less than 0.05 were considered significant. No randomization or blinding was performed.

### Study approval.

Mice were housed and manipulated according to INSERM guidelines and European Directive 2010/63/UE in the local animal care facility (agreements A 31 555 04 and C 31 555 07). Protocols were approved by the French Ministry of Research after review by the local ethical committee (comité d’éthique en expérimentation animale de l’UMS006/CREFRE, CEEA122, Toulouse, France).

## Author contributions

ER, GT, JD, JPC, CP, EM, and DL participated in research design. ER, GT, JD, CB, FB, SCS, MAM, AB, LM, CF, ML, and EM conducted experiments. ER, GT, JD, SCB, CP, EM, and DL performed data analysis and interpretation. ER, JD, CP, and DL drafted the paper. RD, PDD, and CM provided useful advice and edited the paper. CP, EM, and DL supervised the studies. All authors read and approved the final manuscript.

## Supplementary Material

Supplemental data

## Version 1 01/18/2022

In-Press Preview

## Version 2 02/22/2022

Electronic publication
