# Supplementary material for: ChREBPβ is dispensable for the control of glucose homeostasis and energy balance
Source: JCI Insight. 2022 Feb 22;7(4):e153431. doi: 10.1172/jci.insight.153431 (PMC8876429; doi:10.1172/jci.insight.153431)
Supplement: Supplemental data [file jciinsight-7-153431-s175.pdf]

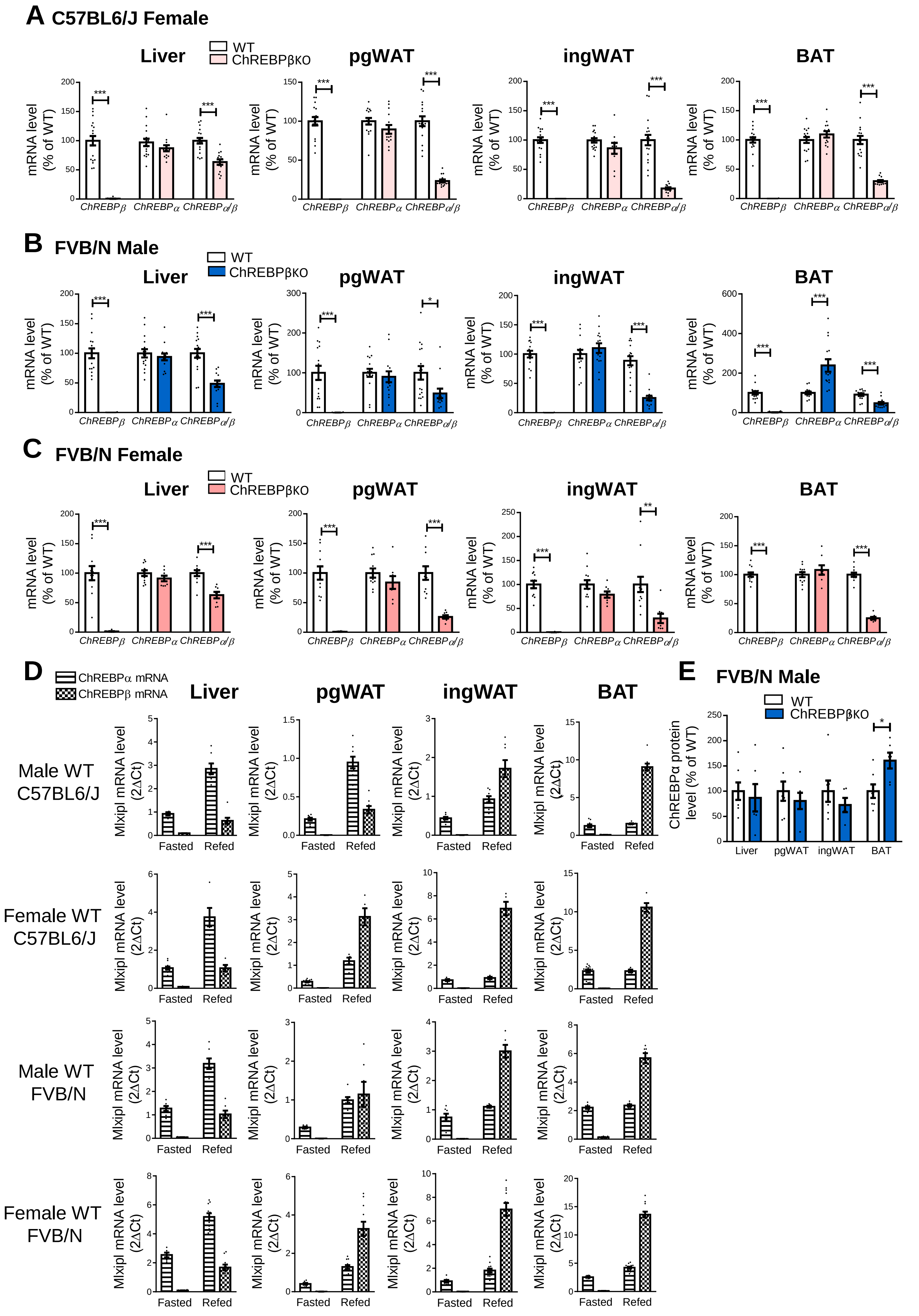

A C57BL6/J Female
WT
ChREBPβKO
Liver
pgWAT
ingWAT
BAT
mRNA level (% of WT)
ChREBPβ
ChREBPα
ChREBPα/β
***
B FVB/N Male
WT
ChREBPβKO
Liver
pgWAT
ingWAT
BAT
mRNA level (% of WT)
ChREBPβ
ChREBPα
ChREBPα/β
C FVB/N Female
WT
ChREBPβKO
Liver
pgWAT
ingWAT
BAT
mRNA level (% of WT)
ChREBPβ
ChREBPα
ChREBPα/β
D
ChREBPα mRNA
ChREBPβ mRNA
Liver
pgWAT
ingWAT
BAT
Male WT C57BL6/J
Female WT C57BL6/J
Male WT FVB/N
Female WT FVB/N
Mlxipl mRNA level (2ΔCt)
Fasted
Refed
E FVB/N Male
WT
ChREBPβKO
ChREBPα protein level (% of WT)
Liver
pgWAT
ingWAT
BAT

Figure S1. Generation of ChREBPβ-deficient mice in C57BL6/J and FVB/N genetic backgrounds

(A,B,C) mRNA levels of ChREBP isoforms in liver, perigonadal white adipose tissue (pgWAT), inguinal WAT (ingWAT) and interscapular brown adipose tissue (BAT) of) refed female C57BL6/J (n=14-16) (A), male (n=13-16) (B) and female FVB/N (n=8-11) (C) mice. (D) mRNA levels of the ChREBPα and β isoforms of the *Mlxipl* gene in liver, pgWAT, ingWAT and BAT of wild type fasted and refed male and female C57BL6/J (n=6-15) and FVB/N (n=6-12) mice. Data are represented as $2^{\Delta Ct}$ values. (E) Quantitation of ChREBP protein levels in liver, pgWAT and BAT of refed male FVB/N (n=5-7) mice. Data are mean ± SEM. Statistical analysis was performed using Mann-Whitney tests. *P<0.05, **P<0.01, ***P<0.001.

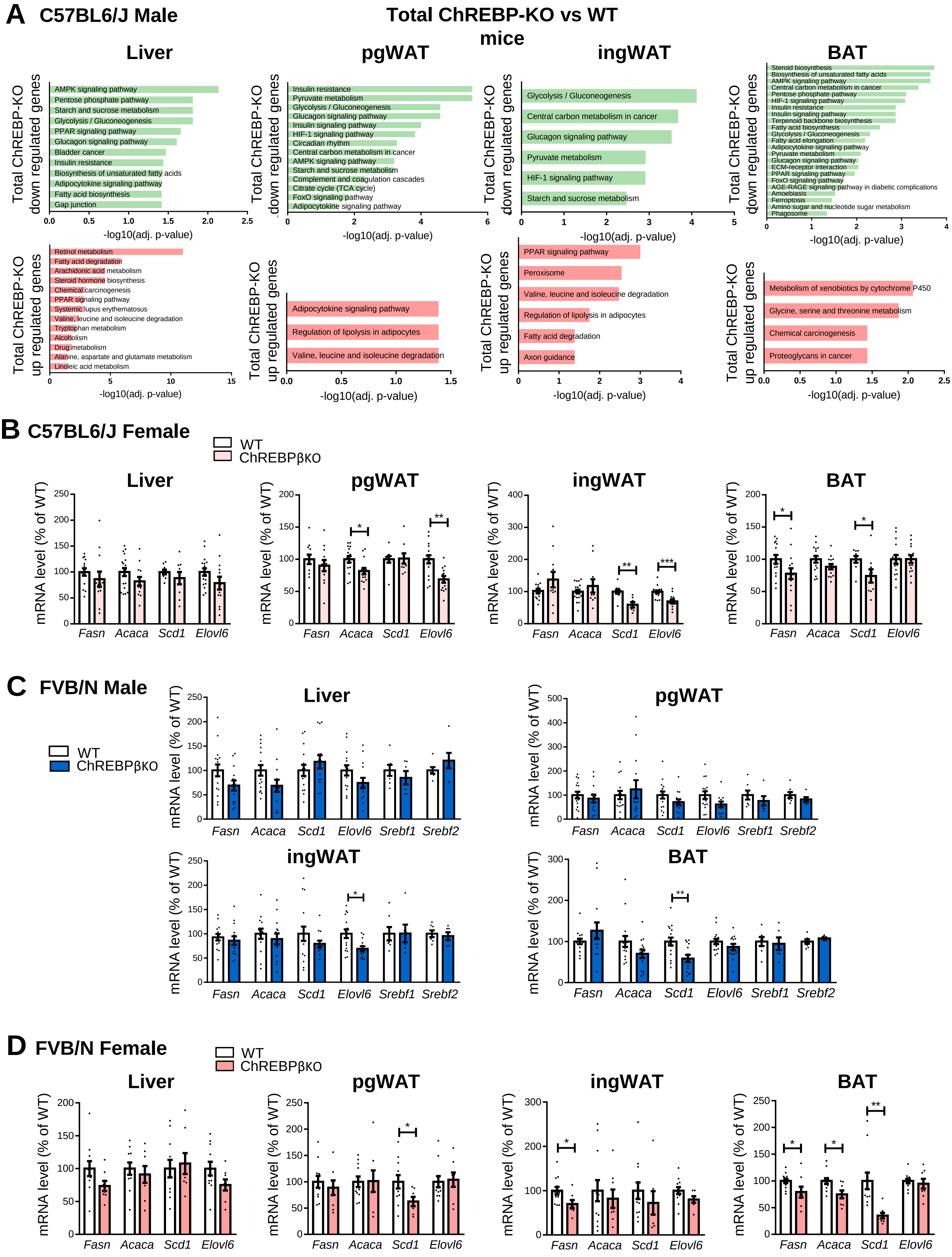
A C57BL6/J Male
Total ChREBP-KO vs WT mice
Liver
pgWAT
ingWAT
BAT
Total ChREBP-KO down regulated genes
AMPK signaling pathway
Pentose phosphate pathway
Starch and sucrose metabolism
Glycolysis / Gluconeogenesis
PPAR signaling pathway
Glucagon signaling pathway
Bladder cancer
Insulin resistance
Biosynthesis of unsaturated fatty acids
Adipocytokine signaling pathway
Fatty acid biosynthesis
Gap junction
-log10(adj. p-value)
Insulin resistance
Pyruvate metabolism
Glycolysis / Gluconeogenesis
Glucagon signaling pathway
Insulin signaling pathway
HIF-1 signaling pathway
Circadian rhythm
Central carbon metabolism in cancer
AMPK signaling pathway
Starch and sucrose metabolism
Complement and coagulation cascades
Citrate cycle (TCA cycle)
FoxO signaling pathway
Adipocytokine signaling pathway
Glycolysis / Gluconeogenesis
Central carbon metabolism in cancer
Glucagon signaling pathway
Pyruvate metabolism
HIF-1 signaling pathway
Starch and sucrose metabolism
Steroid biosynthesis
Biosynthesis of unsaturated fatty acids
AMPK signaling pathway
Central carbon metabolism in cancer
Pentose phosphate pathway
HIF-1 signaling pathway
Insulin resistance
Insulin signaling pathway
Terpenoid backbone biosynthesis
Fatty acid biosynthesis
Glycolysis / Gluconeogenesis
Fatty acid elongation
Adipocytokine signaling pathway
Pyruvate metabolism
Glucagon signaling pathway
ECM-receptor interaction
PPAR signaling pathway
FoxO signaling pathway
AGE-RAGE signaling pathway in diabetic complications
Amoebiasis
Ferroptosis
Amino sugar and nucleotide sugar metabolism
Phagosome
Total ChREBP-KO up regulated genes
Retinol metabolism
Fatty acid degradation
Arachidonic acid metabolism
Steroid hormone biosynthesis
Chemical carcinogenesis
PPAR signaling pathway
Systemic lupus erythematosus
Valine, leucine and isoleucine degradation
Tryptophan metabolism
Alcoholism
Drug metabolism
Alanine, aspartate and glutamate metabolism
Linoleic acid metabolism
Adipocytokine signaling pathway
Regulation of lipolysis in adipocytes
Valine, leucine and isoleucine degradation
PPAR signaling pathway
Peroxisome
Valine, leucine and isoleucine degradation
Regulation of lipolysis in adipocytes
Fatty acid degradation
Axon guidance
Metabolism of xenobiotics by cytochrome P450
Glycine, serine and threonine metabolism
Chemical carcinogenesis
Proteoglycans in cancer
B C57BL6/J Female
WT
ChREBPβKO
Liver
pgWAT
ingWAT
BAT
mRNA level (% of WT)
Fasn
Acaca
Scd1
Elovl6
C FVB/N Male
WT
ChREBPβKO
Liver
pgWAT
ingWAT
BAT
mRNA level (% of WT)
Fasn
Acaca
Scd1
Elovl6
Srebf1
Srebf2
D FVB/N Female
WT
ChREBPβKO
Liver
pgWAT
ingWAT
BAT
mRNA level (% of WT)
Fasn
Acaca
Scd1
Elovl6

Figure S2. Gene expression profiles in ChREBP-deficient mice

(A) Pathway analyses from DNA microarray data in liver, perigonadal white adipose tissue (pgWAT), inguinal WAT (ingWAT) and interscapular brown adipose tissue (BAT) of refed C57BL6/J male mice deficient in ChREBPα and β isoforms (Total ChREBP KO) (n=8 per group). (B,C,D) mRNA levels of de novo lipogenesis genes in liver, pgWAT, ingWAT and BAT of refed C57BL6/J female (n=9-18) (B), FVB/N male (n=6-16) (C) and female (n=8-12) (D) refed mice with ChREBPβ deficiency. Data are mean ± SEM. Statistical analysis was performed using Mann-Whitney tests. *P<0.05, **P<0.01, ***P<0.001.

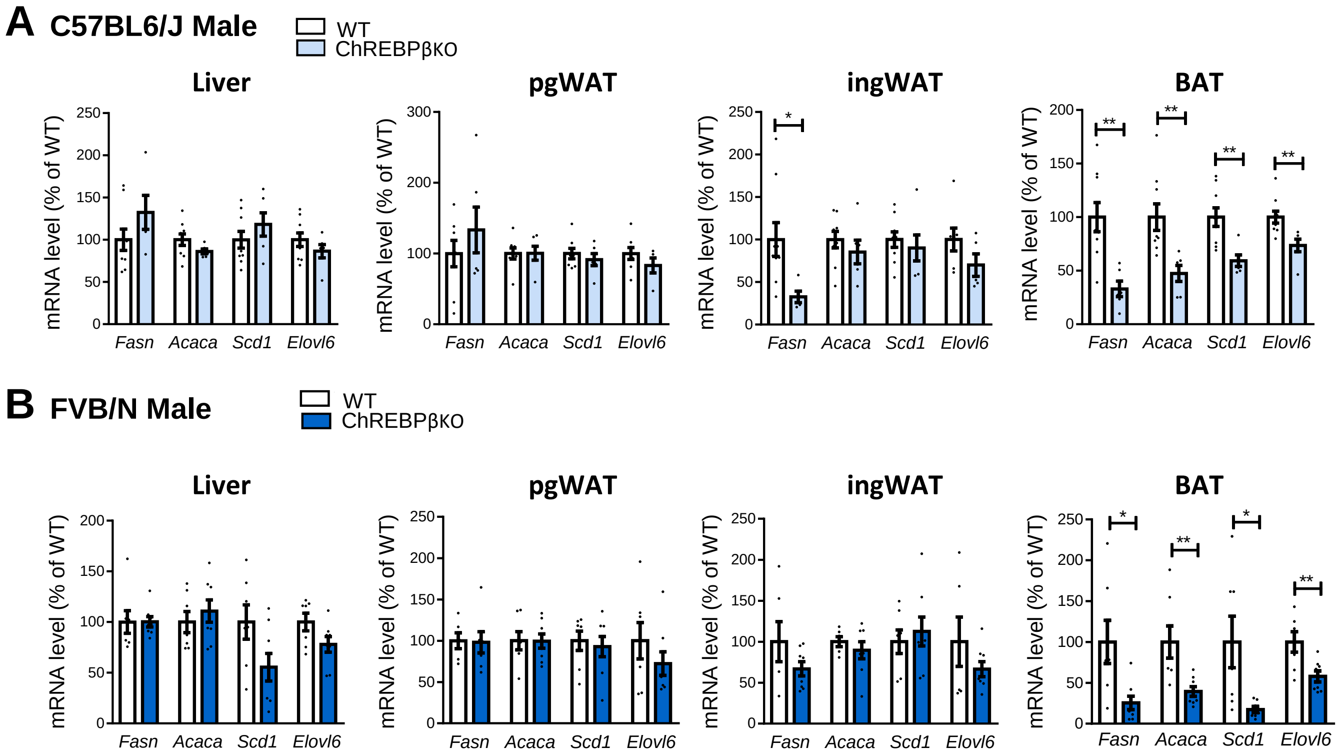

Figure S3. Gene expression profiles in fasted ChREBP-deficient mice

(A,B) mRNA levels of de novo lipogenesis genes in liver, pgWAT, ingWAT and BAT of C57BL6/J (n=5-9) (A) and FVB/N (n=7-8) (B) fasted male mice with ChREBPβ deficiency. Data are mean ± SEM. Statistical analysis was performed using Mann-Whitney tests. *P<0.05, **P<0.01.

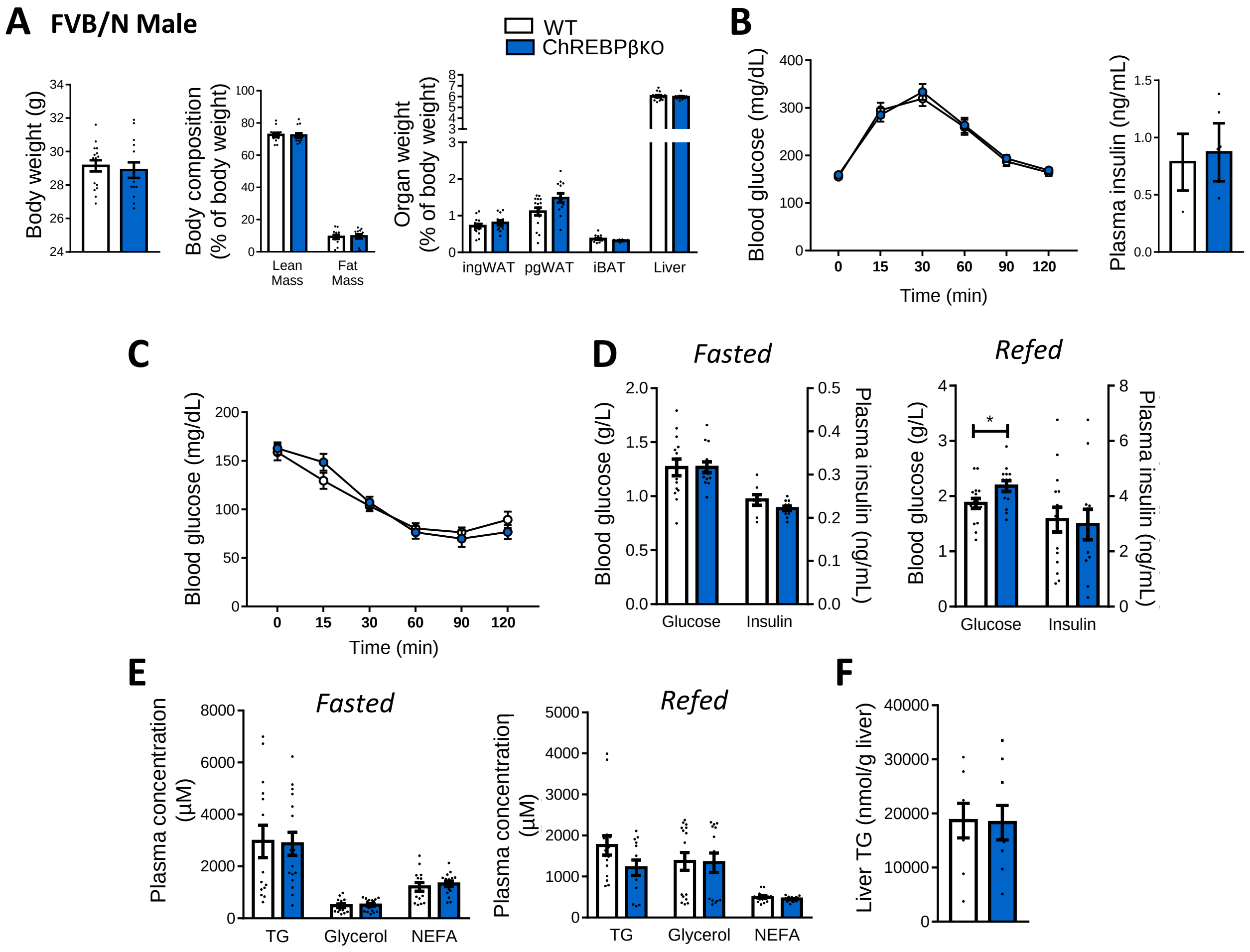

Figure S4. Body composition and glucose homeostasis in male FVB/N ChREBPβ-deficient mice

(A) Body weight, lean and fat mass and, organ weight in refed male FVB/N mice (n=13-16). (B) Glucose tolerance tests and blood insulin levels 15 min post-injection during glucose tolerance tests (n=7-16). (C) Insulin tolerance test (n=7-11). (D) Blood glucose and insulin levels after 24h of fasting (left panels) and 18h of refeeding (right panels) (n=8-16). (E) Blood lipid levels after 24h of fasting (left panels) and 18h of refeeding (right panels). (F) Liver triglyceride (TG) levels after 18h of refeeding (n=8-9). NEFA, non esterified fatty acid. Data are mean ± SEM. Statistical analysis was performed using Mann-Whitney test (A, B, D-F) or two-way ANOVA with Šídák post-hoc tests (B,C). *P<0.05, **P<0.01.

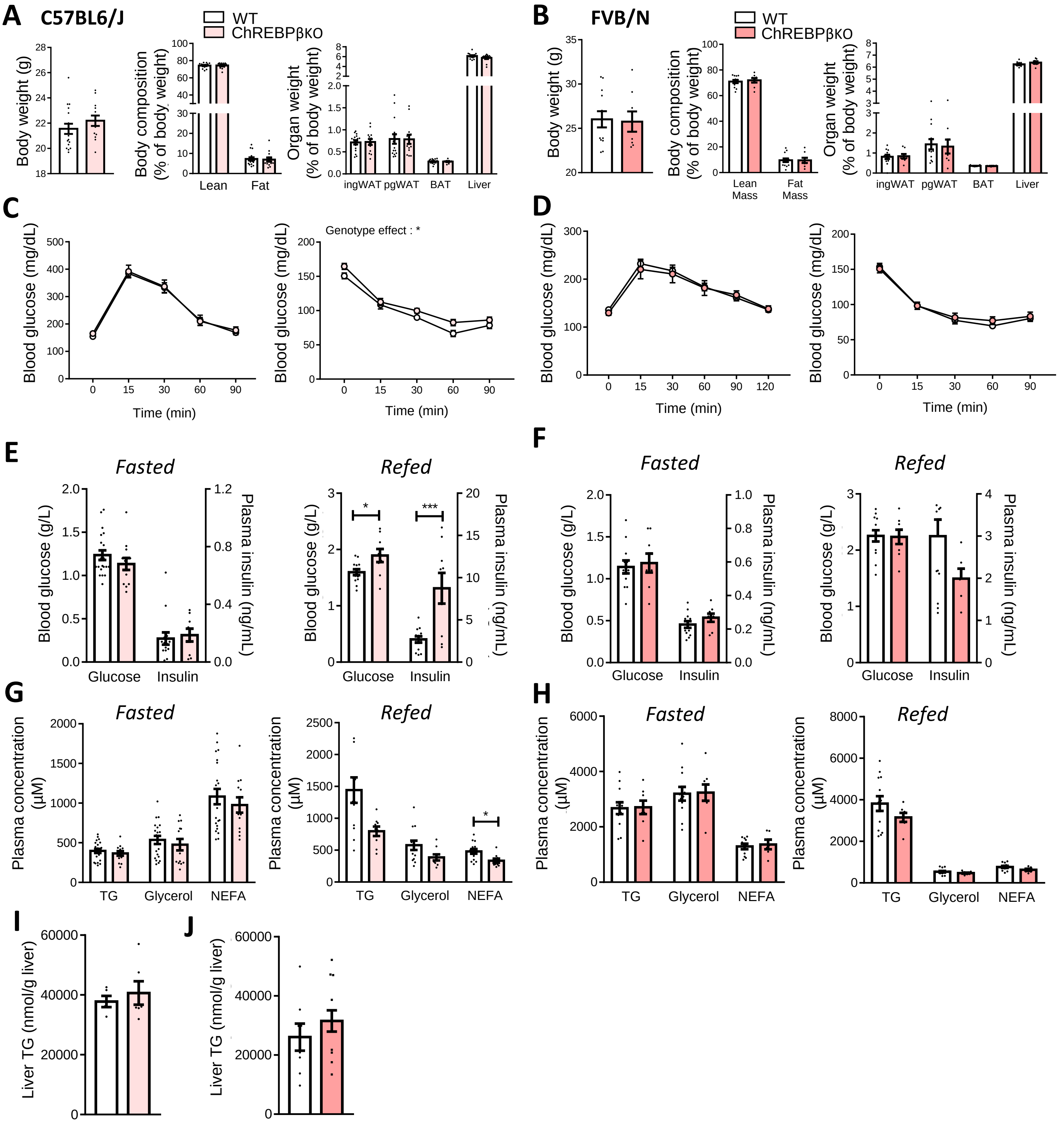

Figure S5. Body composition and glucose homeostasis in female ChREBPβ-deficient mice

(A,B) Body weight, lean and fat mass and, organ weight in refed female C57BL6/J (n=13-18) (A) and FVB/N (n=8-12) (B) mice. (C,D) Glucose (left panels) and insulin (right panels) tolerance tests in female C57BL6/J (n=7-18) (C) and FVB/N (n=10-15) (D) mice. (E-F) Blood glucose and insulin levels after 24h of fasting (left panels) and 18h of refeeding (right panels) in female C57BL6/J (n=9-16) (E) and FVB/N (n=6-13) (F) mice. (G-H) Blood lipid levels after 24h of fasting (left panels) and 18h of refeeding (right panels) in female C57BL6/J (n=9-17) (G) and FVB/N (n=7-13) (H) mice. (I-J) Liver triglyceride (TG) levels in female C57BL6/J (n=5-6) (I) and FVB/N (n=8-12) (J) mice after 18h of refeeding. NEFA, non esterified fatty acid Data are mean ± SEM. Statistical analysis was performed using Mann-Whitney test (A, B, E-J) or two-way ANOVA with Šídák post-hoc tests (C,D). *P<0.05, **P<0.01, ***P<0.001.

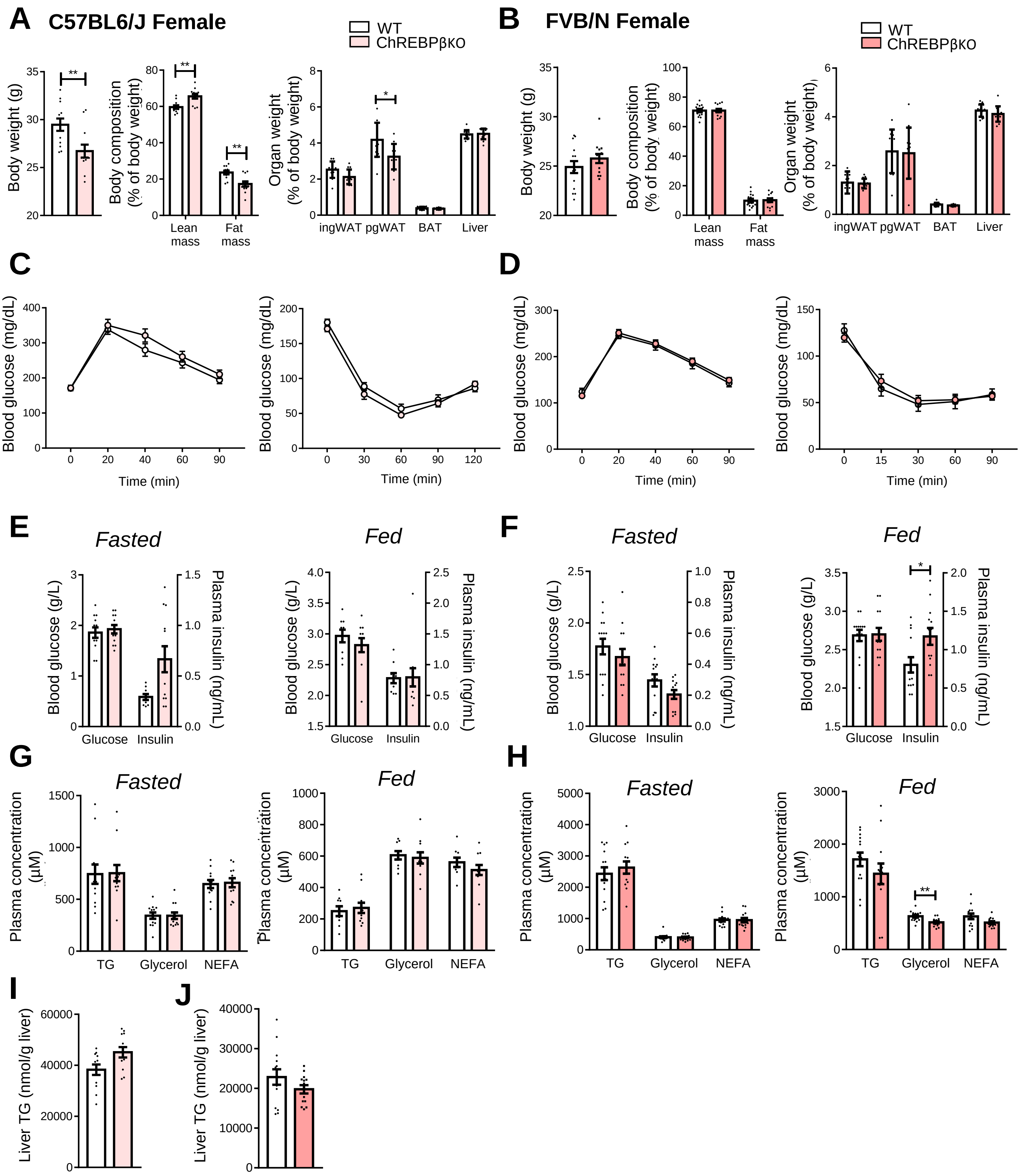

Figure S6. Response of female ChREBPβ-deficient mice to high fat high sucrose diet

(A-B) Body weight, lean and fat mass and, organ weight in C57BL6/J (n=12) (A) and FVB/N N (n=7-14) (B) mice fed high fat high sucrose diet. (C-D) Glucose (left panels) and insulin (right panels) tolerance tests in C57BL6/J (n=12) and FVB/N (8-14) mice. (E-F) Blood glucose and insulin levels after 24h of fasting (left panels) and 18h of refeeding (right panels) in C57BL6/J (n=9-12) (E) and FVB/N mice (n=14-13) (F). (G-H) Blood lipid levels after 24h of fasting (left panels) and 18h of refeeding (right panels) in C57BL6/J (n=9-12) (E) and FVB/N mice (n=14-13) (H). (I-J) Liver triglyceride (TG) levels in C57BL6/J (n=12) (I) and FVB/N (n=13-14) (J) mice. NEFA, non esterified fatty acid. Data are mean ± SEM. Statistical analysis was performed using Mann-Whitney tests (A, B, E-J) or two-way ANOVA with Šídák post-hoc tests (C, D). *P<0.05, **P<0.01..

Supplemental Table 1. ChREBPβ deficiency through CRISPR-Cas9-mediated gene editing technology

(A) Mice born after electroporation of SpyCas9 complexed to two single guide RNAs

Genetic strain	Nb of F0	Nb of mutated F0	Nb of mutated F0 with a single deletion > 500 bp	% mice of interest / F0
C57BL/6J	8	6	4	50
FVB/N	51	20	16	31
Both strains	59	26	20	34

(B) Numbers of litters and pups according to genotypes

Strain	Line	Nb of litters	Nb of pups	Nb of pups per litter	WT (nb/%)	Hemizygous (nb/%)	KO (nb/%)
C57BL/6J	32	42	313	7.5	80 / 26	147 / 47	86 / 27
	52	18	129	7.2	33 / 26	44 / 34	52 / 40
	Both lines	*60*	*442*	*7.4*	*113 / 26*	*191 / 43*	*138 / 31*
FVB/N	15	38	363	9.6	100 / 27	170 / 47	93 / 26
	34	4	30	7.5	11 / 38	13 / 43	6 / 20
	Both lines	*42*	*393*	*9.4*	*111 / 28*	*183 / 47*	*99 / 25*

Mice were from F2 generation and above were derived from matings of heterozygous parents. WT, wild type mice; KO, ChREBPβ-knock-out mice.

Supplemental Table 2. List of genes regulated in brown adipose tissue of ChREBPβ-deficient mice compared to brown adipose tissue of ChREBPα and β-deficient mice

Common ChREBPα and β Up-regulated	ChREBPβ-specific Up-regulated	Common ChREBPα and β Down-regulated	ChREBPβ-specific Down-regulated
Adra1d	*Adrb3*	*Pgm3*	*AK086179*
AK163904	*C1qtnf7*	*Maff*	*A530010L16Rik*
Armcx3	*Enc1*	*Recql4*	*Tmprss9*
Baalc	*Epdr1*	*Dcaf6*	*AK047114*
Car14	*Espn*	*Cbfa2t3*	*Oprl1*
Car3	*Fam124a*	*Acot1*	*BC049352*
Cbr3	*Fbxw4*	*Fras1*	*Sik1*
Ccdc3	*Fgf1*	*Tjp3*	
Cd274	*Mutyh*	*Glul*	
Chst11	*Prrt4*	*Prss35*	
Efemp1	*Rspo4*	*Cldn22*	
Esrrb	*Tnk2*	*Cnst*	
Fam20c	*Trem3*	*Hemk1*	
Fam222a	*Trib3*	*Pdzk1ip1*	
Fzd4	*Trim80*	*Kit*	
Gm14964	*Tuba1a*	*Dmrtc1a*	
Gm6756	*Tuba8*	*Pth1r*	
Gm8096	*Zfp36l2*	*Tspan18*	
Grb14		*AK158867*	
Gss		*C81189*	
Hipk3		*Oxtr*	

Krt79	*Nudt4*
Lgr6	*Fam57b*
Lhfpl2	*Atp1a3*
Lppr4	*Ttc25*
Lrtm1	*BB211547*
Neat1	*Cpeb2*
Ninl	*Gm9112*
Nnmt	*Slc25a1*
Npr3	*Adcy10*
Nr0b1	*Sorl1*
Nrxn2	*Map6*
Phgdh	*Mlxipl*
Plekhg6	*Rhob*
Pon1	*Atp8a1*
Prr32	*Tekt1*
Rnf182	*Gm5126*
Ryr2	*March3*
S100b	*Slc27a1*
Sesn2	*Ccdc57*
Slc40a1	*Hyls1*
Smyd4	*Mc5r*
Sncg	*Prkab1*
Trim67	*Egln3*
Vipr2	*AK037550*
	Tkt

Slc38a10
Gata6
Fabp5
Acsl5
Hmmr
Rgs16
Flrt1
Mapk8ip1
Fhdc1
Foxred2
Tmie
Aspg
Smoc1
Vps37d
Dock3
E130101M22
Focad
Ccdc92
Sqle
Phyhip

Supplemental Table 3. List of primers used in reverse transcription-quantitative PCR

Gene name	Forward primer (5'→3')	Reverse primer (5'→3')
*Mlxip*l(ChREBPα/β)	CACTCAGGGAATACACGCCTAC	ATCTTGGTCTTAGGGTCTTCAGG
Mlxipl(ChREBPα)	CGACACTCACCCACCTCTTC	TTGTTCAGCCGGATCTTGTC
Mlxipl(ChREBPβ)	TCTGCAGATCGCGTGGAG	CTTGTCCCGGCATAGCAAC
Fasn	GCTGCGGAAACTTCAGGAAAT	AGAGACGTGTCACTCCTGGACTT
Acaca	GCCTCTTCCTGACAAACGAG	TGACTGCCGAAACATCTCTG
Scd1	TACACCTGCCTCTTCGGGAT	GCCGTGCCTTGTAAGTTCTG
Elovl6	TGCAGGAAAACTGGAAGAAGTCT	ATGCCGACCACCAAAGATAAA
Srebf1	GCATGCCATGGGCAAGTAC	TGTTGCCATGGAGATAGCATCT
Srebf2	CTCCTTTCTGCCTCTGGTTCTC	GCAAAATGCTCTCTGGTGCAA
Ucp1	CCTGCCTCTCTCGGAAACAA	TGTAGGCTGCCCAATGAACA
Prdm16	CAGCACGGTGAAGCCATTC	GCGTGCATCCGCTTGTG
Cidea	ATCACAACTGGCCTGGTTACG	TACTACCCGGTGTCCATTTCT
Cpt1b	CCGGAAAGGTATGGCCACTT	GAAGAAAATGCCTGTCGCCC
Cox5b	GCTGCATCTGTGAAGAGGACAAC	CAGCTTGTAATGGGTTCCACAGT
Cox8b	TGCGAAGTTCACAGTGGTTC	TCAGGGATGTGCAACTTCA
Hprt	TGGCCATCTGCCTAGTAAAGC	GGACGCAGCAACTGACATTTC
Cox1	ACTATACTACTACTAACAGACCG	GGTTCTTTTTTTCCGGAGTA
Ppia	ACACGCCATAATGGCACTGG	CAGTCTTGGCAGTGCAGAT
